# Accuracy of remote diagnosis of oral mucositis in children and adolescents with cancer using a mobile App: a pilot study

**DOI:** 10.1590/1807-3107bor-2026.vol40.044

**Published:** 2026-07-31

**Authors:** Thayana Maria Navarro Ribeiro de Lima Martins, Paula Maria Maracajá Bezerra, Fábio Gomes dos Santos, Eliane Batista de Medeiros Serpa, Ana Maria Gondim Valença, Simone Alves de Sousa

**Affiliations:** (a) Universidade Federal da Paraíba – UFPB, Graduate Program in Dentistry, João Pessoa, PB, Brazil.; (b) Universidade Federal da Paraíba – UFPB, Graduate Program in Family Health, João Pessoa, PB, Brazil.

**Keywords:** Remote Patient Monitoring, Teledentistry, Mobile Applications, Diagnosis, Oral, Pediatric Dentistry

## Abstract

Remote examinations have become increasingly common in Dentistry, especially with the use of applications. This preliminary cross-sectional study evaluated the accuracy of remote examination of oral mucositis (OM) and severe oral mucositis (SOM) using photos obtained through the Telepediatric Dentistry in Oncology (TON) application, comparing its results with the in-person clinical examination (gold standard). A total of 21 pediatric patients (13 boys, 8 girls; mean age 9.05 ± 3.46 years) undergoing chemotherapy were examined, totaling 28 in-person examinations and 224 photographic images assessed remotely by four calibrated dentists (Kappa > 0.7). The photos covered eight oral cavity sites (lips, right and left buccal mucosa, tongue dorsum, lateral tongue, palate, and floor of the mouth) and were analyzed for the presence or absence of OM and SOM. Among the 28 examinations, 11 presented OM and 3 presented SOM. The remote examination identified all positive cases (sensitivity 100%; 95%CI: 0.741–1), while specificity was 59% for OM, reflecting the occurrence of false positives, especially in the lips and buccal mucosa. Agreement between remote examiners and the gold standard ranged from 0.17 to 1.0, depending on the region and evaluator, being higher in the tongue dorsum, lateral tongue, and floor of the mouth. The results indicate that remote examination via TON may be feasible and has the ability to detect OM and SOM, potentially serving as a complementary tool in dental monitoring of children and adolescents undergoing oncology treatment, particularly when in-person examination is not possible.

## Introduction

Access to dental services is a determinant of quality of life and well-being. Teledentistry has emerged as a promising strategy to reduce inequalities, improve clinical outcomes, and support care.^1^ It involves applying information and communication technologies (ICTs) in dentistry, enabling the exchange of clinical information, patient care, and implementation of distance education strategies.^2^ These teledentistry models can act as a complement to traditional approaches or as an alternative in contexts where in-person care is not feasible.^3^

A systematic review with meta-analysis demonstrated that teledentistry performs satisfactorily in caries diagnosis, although with heterogeneity among studies.^4^ The CariesCare International protocol was applied to images obtained remotely, both with professional cameras and smartphones, demonstrating the feasibility of teledentistry in different care contexts.^5^

The use of photographs in teledentistry has proven to be a viable strategy for conducting oral examinations and screening dental treatments, even in older adult patients^6^. However, despite promising results, larger studies are still needed to enable broader generalization of findings.^6^

The use of teledentistry tools to detect oral lesions can result in benefits such as early diagnosis, appropriate treatment, and closer monitoring of potentially malignant disorders, in addition to representing a viable alternative to in-person dental consultations for lesion identification and differential diagnosis. Proper implementation of these tools can potentially expand access to dental care.^7^

A study^8^ on the implementation of the MeMoSA^®^ application in 2021 indicated this tool as a facilitator in accurate identification and referral of oral cancer cases, reducing unnecessary patient exposure in contexts such as the COVID-19 pandemic^8,9^Also during the COVID-19 pandemic, the need for remote methods became critical to support oncology services, as hospital visits were limited to new treatment cycles or medical emergencies.^10^

A study by Mola et al.^11^ indicated that teledentistry is a viable alternative to in-person clinical diagnosis in pediatric dental consultations, showing similar accuracy in detecting caries and dental anomalies in children, as well as in defining therapeutic planning. Based on these potential applications, the Teleinterconsulta in Stomatology application in Paraía was developed to enable interaction between dentists in health services and specialists capable of assisting in the diagnosis of routine clinical cases, supporting oral and maxillofacial lesions, and speeding up oral healthcare services in the state of Paraíba, Brazil.^12^

Such tools have proven useful regarding the diagnosis and management of most pediatric dental emergencies, and it has been observed that less experienced users can increase their confidence in treatment guidance via teledentistry by seeking more detailed information during consultations.^13^ A systematic review evaluated the impact of teledentistry on access to oral health services, concluding that the tool improves access, facilitates early diagnosis, and can benefit underserved populations.^14^

From this perspective, integrating teledentistry tools, artificial intelligence, and hybrid dental practice models can improve access and equity in oral health, provided there is investment, support, and collaboration.^15^ The knowledge and experiences of professionals need to be considered in policies and practices for patient care.^16^ Research involving teledentistry is urgent to assess its impact on oral health equity, as it is already known that teledentistry can improve clinical outcomes.^17^

Thus, the Telepediatric Dentistry in Oncology (TON) application was developed for teledental monitoring of children and adolescents with cancer, as well as to provide oral health education information and enable patient follow-up through the submission of oral cavity photographs.^18^ The present study aimed to evaluate the accuracy of photos obtained through the TON application for remote examination of oral mucositis, comparing its results with the in-person clinical examination.

## Methods

### Study type

**Figure 1:**
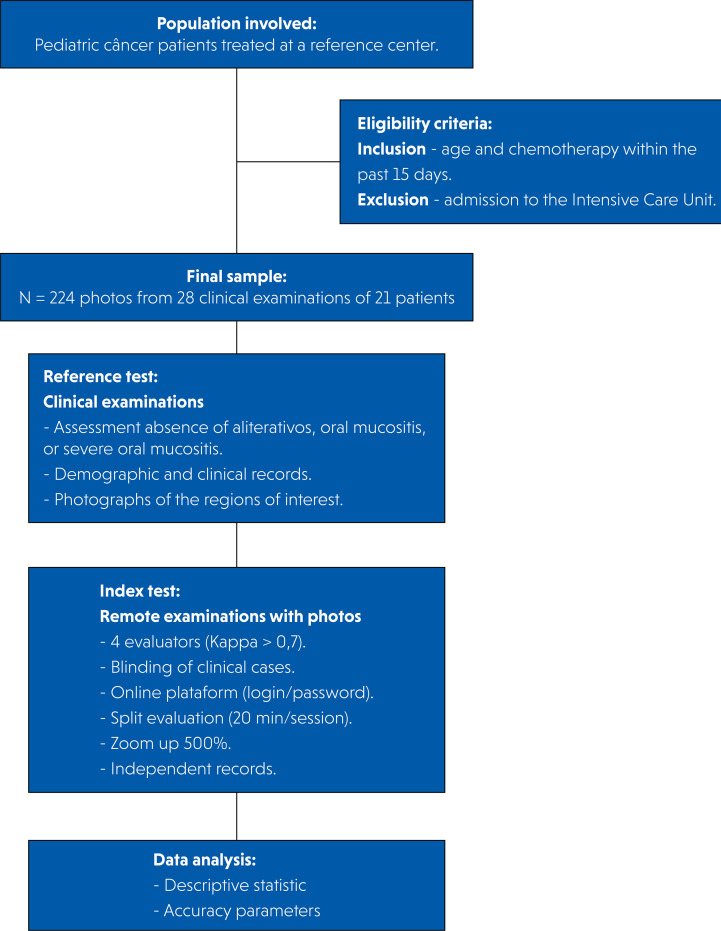
Schematic diagram illustrating the study design.

A preliminary cross-sectional diagnostic accuracy study^19^ was conducted between July and September 2023, aiming to compare remote examinations of oral mucositis with in-person examinations. The writing was guided by the criteria outlined by the Quality Assessment of Diagnostic Accuracy Studies (QUADAS-2)^20^ and the Standards for Reporting Studies of Diagnostic Accuracy (STARD) (Figure 1).^21^

### Study setting

The study was conducted in the pediatric oncology department of xxx. HNL is one of the High Complexity Oncology Centers (CACON) in the city, providing care to individuals aged 0 to 19 years, with an average of 159 new pediatric patients admitted annually.^22^

### Population and sample

As this study was carried out in a reference center for pediatric cancer, the sample of this pilot study was non-probabilistic and convenient due to the rarity and specificity of the population studied. Participants were selected based on practical accessibility to pediatric patients/caregivers who met the profile of app users. Thus, individuals undergoing chemotherapy in the last 15 days, aged 4 years or older, and not hospitalized in the HNL Intensive Care Unit (ICU) during data collection were included. This careful selection aimed to ensure the minimum necessary collaboration for the examinations, while simultaneously guaranteeing biosafety and clinical stability of the patients, as those in the ICU had more severe conditions. The analysis unit adopted was the clinical examination, considering the limited population and the possibility that the same patient could participate more than once in the study, provided that they were in distinct chemotherapy cycles, which enabled a higher number of examinations.

### Data collection

#### Researcher training

Prior to data collection, five examiners (one for in-person examinations and four for remote examinations) participated in synchronous virtual meetings to standardize their assessment of likely conditions in the oral cavity of children and adolescents. Photographic images of Oral Mucositis (OM) and Severe Oral Mucositis (SOM) in various oral sites were used to help standardize the examiners’ evaluations.

OM was defined as mucosal changes with erythema, desquamation, hyperkeratinization, or epithelial erosion without connective tissue exposure, and SOM as the presence of ulcerations. Diagnosis was standardized in two stages: presence/absence of OM and presence/absence of SOM.

#### In-person examinations

These examinations were considered the reference test or gold standard. The presence or absence of OM and SOM was evaluated using the Modified Oral Assessment Guide (OAG)^23^. The sole examiner for this stage was calibrated for the OAG (Kappa > 0.7).

The OAG classifies the severity of oral changes based on the evaluation of different oral sites, including lips, mucosa, tongue, gingiva, and saliva, as well as the presence of pain during oral functions such as speech and swallowing. Each parameter is scored from 1 to 3, with 1 considered normal, 2 indicating mild/moderate changes, and 3 indicating severe changes. The sum of the scores determines the overall degree of oral involvement, reflecting the severity of mucositis.^23^

Eight sites of the patients’ oral cavity were analyzed in a fixed sequence, and each examination was considered an independent analysis unit. These sites were the lips, right buccal mucosa, left buccal mucosa, tongue dorsum, right lateral tongue, left lateral tongue, palate, and floor of the mouth.

All examinations were performed under artificial lighting (headlamp) and using tongue retractors. Each examination was assigned a unique code, and the data were recorded on a standardized form. Personal data of the children and adolescents were also collected, including age, sex, type of cancer, history of OM and/or SOM, and date of the last chemotherapy session.

#### Photographic records in the TON application

**Figure 2:**
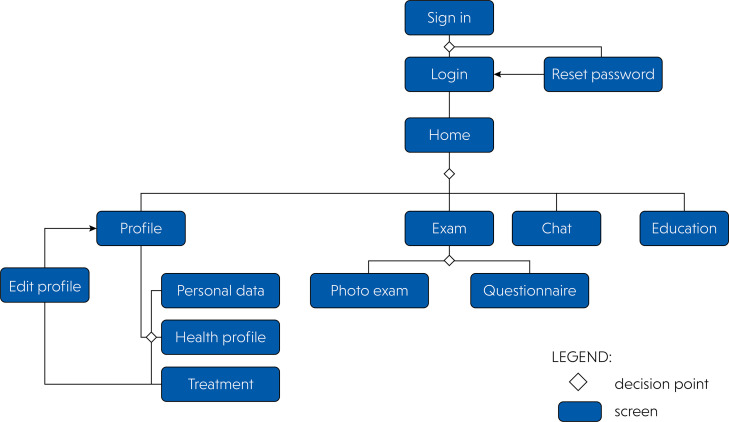
Flowchart illustrating the functioning of the TON application.

The Telepediatric Dentistry in Oncology (TON) is an application available for mobile phones and computers designed for children and adolescents with cancer, particularly to support oral healthcare for patients who are geographically distant from oncology treatment centers. The workflow of the application is shown in Figure 2, following interface adjustments made by graphic design professionals.^18^ When used on a device, the TON examination feature automatically activates the camera flash, allowing image quality adjustment with the aid of the provided lighting. The system additionally includes a “point” guide that facilitates focus during capture.

**Figure 3:**
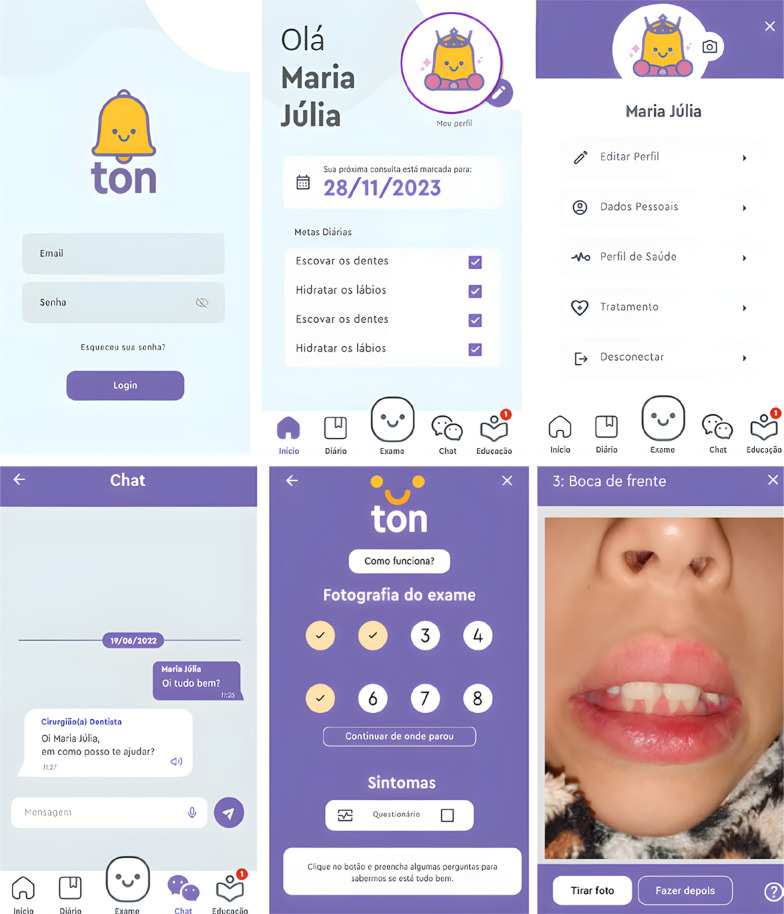
Screen interfaces of the TON application.

Photographs in this stage were taken focusing only on the analysis sites, thereby ensuring participants’ anonymity. All photos were captured within the examination screen of the application (Figure 3) and were taken using a single TON user, designated “Pilot,” by the same examiner calibrated for the OAG (Kappa < 0.7) who conducted the in-person examinations. The purpose was to test the application’s photographic tool and the remote examination questionnaire, which is provided immediately after the examination (on the screen, it is called “Symptoms”). This questionnaire includes four objective questions regarding oral pain, diet, xerostomia, and lip dryness.

Moreover, all photographs were taken using a single mobile phone, Moto G Pure, with a 13-megapixel camera and a resolution of 4163x3122 pixels, on the examination screen of the TON application. All photographs were obtained following the same sequence of regions, always starting with the lips and ending with the floor of the mouth (photos taken from 1 to 8), as shown in Figure 3. After being captured, the photos were uploaded to the TON monitoring website where they could be accessed during remote examinations.

#### Remote examinations

The remote examinations were analyzed in this stage, which were considered the index test. After coding and accessing the TON application website, the photographs were evaluated remotely by four independent dentists, calibrated for the OAG (Kappa > 0.7) and experienced in treating patients undergoing oncology therapy. These researchers had no prior knowledge of the clinical conditions photographed, ensuring impartiality in performing remote diagnoses.

The platform used for the index test was the application control website (https://telodon.vercel.app/login), accessed exclusively via login and password as it is a protected server, in accordance with the General Data Protection Law.^24^ All access was performed on laptops (with 14 to 15.6-inch screens). The photo evaluation was conducted in segments, always reviewing the images after they were uploaded on the same day as the in-person clinical examination.

A maximum continuous analysis time of 20 minutes per session was adopted to prevent visual saturation of the remote examiners. In addition, images could be enlarged up to the maximum limit of the Google Chrome browser (500%), which was the standard set for site access. Each remote evaluator recorded their data in an independent spreadsheet, and the remote examinations followed the same OM and SOM criteria as the reference tests, considering the presence or absence.

## Data analysis

Using the in-person clinical examination as the gold standard, the frequencies of true positives, false positives, false negatives, and true negatives were determined per examination (n = 28) and for each individual site evaluated. Accuracy measures of the remote examination were also calculated, including sensitivity, specificity, positive predictive value, and negative predictive value for each clinical outcome (Oral Mucositis or Severe Oral Mucositis) and for each region of interest. Accuracy calculations were performed using Excel^®^ 2018 after data tabulation.

Confidence intervals (95%) for the accuracy measures were calculated using a contingency table comprising true positives (TP), false negatives (FN), true negatives (TN), and false positives (FP). Analyses were conducted in RStudio (R Foundation for Statistical Computing, Vienna, Austria), version 4.2.1, using the binom package and the Wilson method for estimating confidence intervals for proportions.

Agreement between the evaluators and the gold standard was assessed using Cohen’s Kappa coefficient. This index was obtained by comparing the binary classifications (presence/absence of the condition) provided by each examiner and the gold standard. The Kappa value and its 95% confidence interval were calculated for each evaluation. Then, the mean Kappa values were computed, considering all assessments for each clinical outcome for each examiner in relation to the gold standard. All calculations were performed in RStudio with the assistance of the irr and psych packages, including determining the confidence interval for the mean.

Absolute agreement (100%) was observed between evaluations in some cases, resulting in no variability in responses. Thus, the Kappa coefficient could not be estimated in these situations, and the simple agreement index was used instead, expressed as the proportion of classifications that coincided between the examiner and the gold standard. Analyses were initially conducted in spreadsheets (Excel^®^), then processed in RStudio and confirmed in Jamovi software (version 2.3.21.0: The Jamovi Project, Sydney, Australia) using the Interrater Reliability module.

Additionally, when necessary, the approximate confidence interval (CI) of the mean was obtained from the arithmetic mean of the lower and upper limits of individual CIs per examiner and/or site. First, the means of the lower limits were calculated, followed by the means of the upper limits, resulting in the final estimate of the approximate CI of the mean. The sample power was calculated a posteriori in R for 28 participants at a 5% significance level, ranging from 84.9% to 98.8%, consistently above the 80% threshold.

## Ethical considerations

The study was approved by the Ethics Committee of the Health Sciences Center at the Federal University of Paraíba (UFPB) and was conducted in accordance with the Declaration of Helsinki (CAAE 50161921.7.0000.5188, opinion number 4.992.306). All photographs were taken exclusively focusing on the region of interest for analysis, without capturing any other patient features. The platform used is password-protected and accessible only to the researchers involved. Participant data remain confidential, and the caregivers of the users also authorized the exclusive use of the photographs for research purposes through signing the Informed Consent Form (ICF). Pediatric patients also formally provided individual authorization via the Assent Form.

## Results

### Sample characteristics

A total of 21 patients were examined in person, seven of whom were examined during two different chemotherapy cycles, totaling 28 in-person clinical examinations. In turn, 224 photographic images were evaluated remotely, as eight areas of the oral cavity were examined per session, resulting in 28 remote examinations.

All images were considered satisfactory for the index test, as no images were excluded due to technical quality issues such as lack of focus. Three losses corresponded to three incomplete examinations from patients who did not allow completion of photographic records, and one patient did not permit the in-person examination. The mean age of the children and adolescents was approximately 9.05 years, with a standard deviation of 3.46 years. The sample included 13 male individuals (61.9%) and 8 female individuals (38.1%). Prior to the examinations, 4 patients (19%) had no history of oral mucositis. The characteristics of the participants in this preliminary study are summarized in Table 1.

**Table 1:** Characterization of the patients participating in the study.

Patient code	Sex	Diagnosis	Number of cycles during the study	Chemotherapeutic agents used during the study	History OM/SOM
P001	M	ALL	2	Cytarabine, etoposide, vincristine, methotrexate, oncospar.	Yes
P002	F	Non-Hodgkin Lymphoma	2	Methotrexate, mesna, cyclophosphamide.	Yes
P003	M	Hodgkin Lymphoma	2	Mesna, ifosfamide, gemcitabine, vinorelbine.	Yes
P004	F	ALL	1	Mesna, cytarabine, mercaptopurine	No
P005	F	ALL	1	Vincristine and daunorubicin	No
P006	M	ALL	1	Cyclophosphamide, methotrexate, mesna, thioguanine and cytarabine	Yes
P007	M	Rhabdomyosarcoma	2	Ifosfamide, vincristine, actinomycin and doxorubicin	Yes
P008	F	AML	1	Cytarabine and daunorubicin	Yes
P009	M	ALL	1	Mercaptopurine and methotrexate	Yes
P010	M	ALL	1	Vincristine and daunorubicin	No
P011	F	AML	1	Fludarabine, cytarabine and methotrexate	Yes
P012	M	ALL	1	Vincristine, methotrexate and daunorubicin	Yes
P013	F	ALL	1	Cytarabine, methotrexate and etoposide	Yes
P014	M	Osteosarcoma	2	Etoposide, mesna, ifosfamide, cisplatin, doxorubicin and methotrexate	No
P015	M	ALL	2	Thioguanine, vincristine, methotrexate, ifosfamide, mitoxantrone, daunorubicin and oncospar	Yes
P016	F	Hodgkin Lymphoma	1	Doxorubicin, bleomycin, vinblastine and dacarbazine	Yes
P017	F	Rhabdomyosarcoma	1	Ifosfamide and doxorubicin	Yes
P018	M	ALL	2	Vincristine, methotrexate and cytarabine	Yes
P019	M	Non-Hodgkin Lymphoma	1	Methotrexate, rituximab, etoposide and cytarabine.	Yes
P020	M	ALL	1	Mercaptopurine, vincristine, methotrexate and cytarabine	Yes
P021	M	AML	1	Tretinoin, cytarabine and daunorubicin	Yes

OM: oral mucositis; SOM: severe oral mucositis; M: male; F: female; ALL: acute lymphoblastic leukemia; AML: acute myeloid leukemia.

### Findings of oral mucositis (OM)

**Figure 4:**
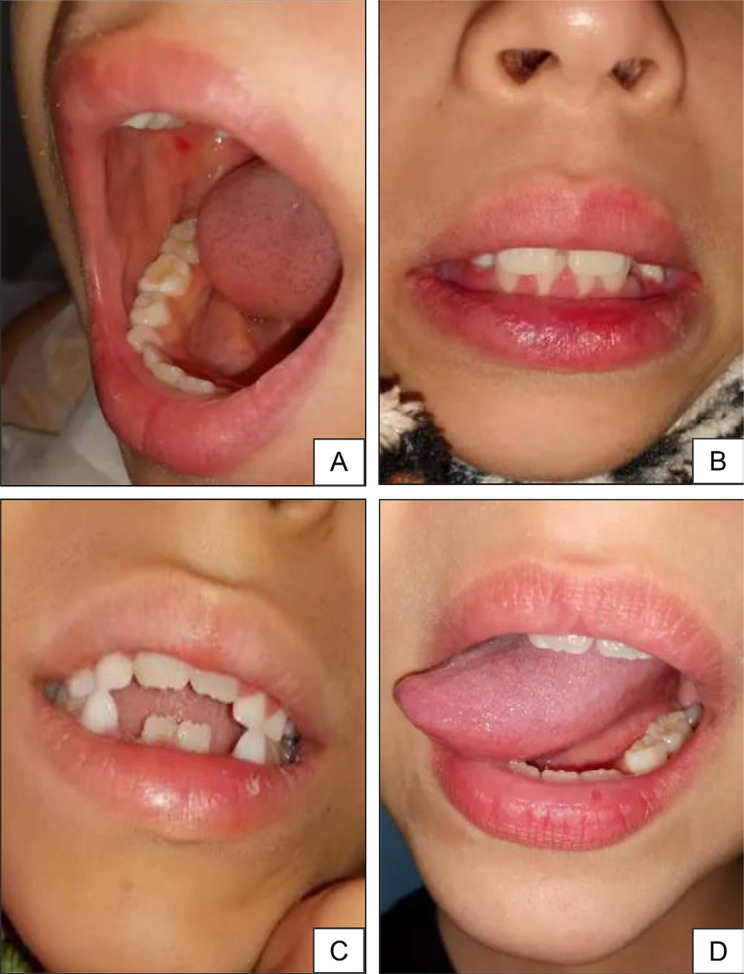
Photographs obtained through the TON app: A-) True Positive (OM - left buccal mucosa region); B-) True Positive (SOM - lip region); C-) False positive (OM - lip region); D-) False positive (OM - right lateral region of the tongue).

Out of the 28 examinations, 11 showed involvement in at least one region with OM (corresponding to the total true positives). Among these OM cases, the remote examination was able to identify all cases consistent with the condition compared to the gold standard (0 false negatives), as indicated in Table 2 and in Additional Tables 1 and 2 available at the link (https://sl1nk.com/supplementarymaterialsaccuracyomandsom). Some cases are illustrated in Figure 4.

**Table 2:** Proportion of true positives, true negatives, false positives, and false negatives for oral mucositis relative to the total number of examinations.

Outcome	TP (%)	FP (%)	TN (%)	FN (%)	Sensitivity (95%CI)	Specificity (95%CI)	Accuracy (95%CI)
OM	11	12	17	0	1	0.59	7000%
	(39.28)	(42.86)	(60.71)	0	(0.74–1)	(0.41–0.74)	(54.6–81.9)
SOM	3 (10.71)	8	25	0	1	0.76	77.8
		(28.57)	(89.28)	0	(0.44–1)	(0.59–0.87)	(61.9–88.3)

OM: oral mucositis; SOM: severe oral mucositis; TP: true positives; FP: false positives; TN: true negatives; FN: false negatives; CI: confidence interval.

The sensitivity of this overall analysis was 100% (95%CI: 0.741–1), indicating that all positive cases according to the gold standard were identified, although the wide confidence interval reflects the sample size limitation of OM cases (n = 11). Specificity was 59% (95%CI: 0.41 to 0.74), with 17 out of 28 negative cases correctly classified, suggesting that false positives may occur (Supplementary Material). These results indicate good ability of the application’s photographs to detect positive cases, but attention should be given to interpreting negative results due to the limited sample size.

The most affected sites by OM were the lips, right buccal mucosa, and left buccal mucosa. The dorsum of the tongue and the floor of the mouth showed no OM lesions (Table 3). Discrepancies were observed in analyzing the results by site between in-person and remote examinations at affected sites. In these cases, remote examinations detected possible OM conditions not visible during in-person examination, resulting in false positives (Table 3).

**Table 3:** Number of correct and incorrect responses, sensitivity, specificity, accuracy, and predictive values (positive and negative) for the outcome OM. Values compiled across examiners.

Area	TP	FP	TN	FN	Sen	Spec	PPV (%)	NPV (%)	Ac (%)
					(95%CI)	(95%CI)			(95%CI)
Lips	6	25	22	0	1.00	0.468	19.35	100	52.83
					(0.61–1)	(0.33–0.61)			(39.7–65.6)
RBM	5	16	20	0	1.00	0.555	23.80	100	60.97
					(0.56–1)	(0.39–0.70)			(45.7–74.3)
LBM	3	18	25	0	1.00	0.581	14.28	100	60.86
					(0.44–1)	(0.43–0.72)			(46.5–73.6)
TD	0	5	28	0	*	0.848	*	100	84.84
						(0.69–0.93)			(69.1–93.3)
RLT	2	7	26	0	1.00	0.787	22.22	100	80
					(0.34–1)	(0.62–0.89)			(64.1–90.0)
LLT	2	4	26	0	1.00	0.866	33.33	100	87.50
					(0.34–1)	(0.70–0.95)			(71.9–95.0)
Palate	1	5	27	0	1.00	0.844	16.66	100	84.84
					(0.21–1)	(0.68–0.93)			(69.1–93.3)
FM	0	10	28	0	*	0.737	*	100	73.68
						(0.58–0.85)			(58.0–85.0)

RBM: right buccal mucosa; LBM: left buccal mucosa; TD: tongue dorsum; RLT: right lateral tongue; LLT: left lateral tongue; FM: floor of the mouth; TP: true positives; FP: false positives; TN: true negatives; FN: false negatives; Sen: sensitivity; Spec: specificity; PPV: positive predictive value; NPV: negative predictive value; Ac: accuracy, *could not be calculated due to the absence of true positives in the sample; CI: confidence interval.

### Findings of severe oral mucositis

Severe oral mucositis (SOM) was identified in fewer cases compared to oral mucositis (OM), totaling three true positives (lips, dorsum of the tongue, and palate). Despite the low occurrence, the images proved useful for identifying all SOM cases, with no false negatives recorded (Table 2). False positives were observed in the lips, right and left buccal mucosa, as well as the dorsum of the tongue (Table 4).

**Table 4:** Number of correct and incorrect responses, sensitivity, specificity, accuracy, and predictive values (positive and negative) for the outcome SOM. Values compiled across examiners.

Area	TP	FP	TN	FN	Sen	Spec	PPV (%)	NPV (%)	Ac (%)
					(95%CI)	(95%CI)			(95%CI)
Lips	1	6	27	0	1.00	0.82	14.28	100	82.35
					(0.21–1)	(0.66–0.91)			(69.5–95.1)
RBM	0	5	28	0	*	0.85	*	100	84.84
						(0.69–0.93)			(72.5–97.1)
LBM	0	1	28	0	*	0.97	*	100	96.55
						(0.83–0.99)			(89.9–100)
TD	1	2	27	0	1.00	0.93	33.33	100	93.33
					(0.21–1)	(0.78–0.98)			(84.4–100)
RLT	0	0	28	0	*	1.00	*	100	100
						(0.88–1)			(88–100)
LLT	0	0	28	0	*	1.00	*	100	100
						(0.88–1)			(88–100)
Palate	1	0	27	0	1.00	1.00	100	100	100
					(0.21–1)	(0.88–1)			(88–100)
FM	0	0	28	0	*	1.00	*	100	100
						(0.88–1)			(88–100)

RBM: right buccal mucosa; LBM: left buccal mucosa; TD: tongue dorsum; RLT: right lateral tongue; LLT: left lateral tongue; FM: floor of the mouth; TP: true positives; FP: false positives; TN: true negatives; FN: false negatives; Sen: sensitivity; Spec: specificity; PPV: positive predictive value; NPV: negative predictive value; Ac: accuracy, *could not be calculated due to the absence of true positives in the sample; CI: confidence interval.

Similar to OM, the small number of cases limits generalization of the findings. However, as this is a preliminary analysis of the TON application’s photographic tool, it can be stated that the remote examination also yielded satisfactory results for detecting more severe cases.

### Concordance analysis

Table 5 presents the concordance analysis between the gold standard and the four remote examiners by region assessed for the OM outcome. The results highlight variations across different evaluated areas, with distinct concordance indices. The “Dorsum of the tongue” (DT) and “Right lateral tongue” (RLT) areas showed the highest concordance rates, while the “Lips,” “Right buccal mucosa” (RBM), and “Left buccal mucosa” (LBM) areas exhibited the lowest.

**Table 5:** Agreement between the gold standard and the four remote examiners by region in OM.

Area	Agreement	Agreement	Agreement	Agreement	Mean Agreement
	PO X EX1 (95%CI)	PO X EX2 (95%CI)	PO X EX3 (95%CI)	PO X EX4 (95%CI)	(95%CI)
Lips	0.48	0.53	0.34	0.81	0.54
	(0.19–0.76)	(0.24–0.83)	(0.09–0.59)	(0.09–1)	(0.35–0.73)
	p = 0.003	p = 0.001	p = 0.0167	p = 0.0000125	
	0.70	0.79	0.36	0.79	0.66
RBM	(0.4–1)	(0.51–1)	(0.09–0.62)	(0.51–1)	(0.34–0.98)
	p = 0.0000958	p = 0.0000193	p = 0.0136	p = 0.0000193	
LBM	0.61	0.84	0.17	0.84	0.62
	(0.23–0.99)	(0.53–1)	(-0.018–0.35)	(0.53–1)	(0.32–0.84)
	p = 0.000448	p = 0.00000712	p = 0.112	p = 0.00000712	
TD	100*	100*	82.14*	100*	95.54*
	(100–100)	(100–100)	(67.9–96.3)	(100–100)	(86.8–100)
RLT	1.00	1.00	0.37	0.63	0.75
	(1–1)	(1–1)	(-0.013–0.76)	(0.17–1)	(0.26–1)
	p ≈ 0.000000121	p ≈ 0.000000121	p = 0.011	p = 0.000325	
LLT	1.00	1.00	0.37	0.78	0.79
	(1–1)	(1–1)	(-0.013–0.76)	(0.37–1)	(0.32–1)
	p ≈ 0.000000121	p ≈ 0.000000121	p = 0.011	p = 0.0000227	
Palate	1.00	1.00	0.24	0.65	0.72
	(1–1)	(1–1)	(-0.15–0.63)	(0.019–1)	(0.15–1)
	p ≈ 0.000000121	p ≈ 0.000000121	p = 0.051	p = 0.000241	
FM	96.3*	100*	71.4*	96.3	90.75*
	(89.3–100)	(100–100)	(54.7–88.1)	(89.3–100)	(78.1–100)
Mean	0.84	0.90	0.42	0.81	0.74
	(0.70–0.99)	(0.78–1.00)	(0.27–0.58)	(0.72–0.90)	(0.65–0.84)

RBM: right buccal mucosa; LBM: left buccal mucosa; TD: tongue dorsum; RLT: right lateral tongue; LLT: left lateral tongue; FM: floor of the mouth; G0: gold standard; Ex1: examiner 1; Ex2: examiner 2; Ex3: examiner 3; Ex4:examiner 4; * = Value calculated by simple agreement, derived from the proportion of correct responses among the evaluators relative to the gold standard due to the absence of true positive cases. Confidence intervals were calculated using the mean limits; C: confidence interval.

Examiners 1, 2, and 4 showed high concordance values in nearly all regions (means between 0.81 and 0.90), whereas examiner 3 consistently demonstrated lower results (mean = 0.42; 95%CI: 0.27 to 0.58), with wide confidence intervals, in some cases including values near zero, such as left buccal mucosa (0.17; 95%CI: –0.018 to 0.35; p = 0.112), lateral tongue (0.37; 95%CI: –0.013 to 0.76; p = 0.011), and palate (0.24; 95%CI: –0.15 to 0.63; p = 0.051). These findings suggest low reproducibility for this examiner in certain regions, negatively impacting the overall mean (0.74; 95%CI: 0.65 to 0.84).

However, areas such as the dorsum of the tongue and the floor of the mouth demonstrated high concordance among all examiners, with means higher than those of the other evaluated sites.

The concordance between the gold standard and the four remote examiners, organized by region assessed for the SOM outcome, is shown in Table 6. The data demonstrate overall concordance closer to 100%, including perfect concordance in some sites and examiners. The areas of “Right lateral tongue” (RLT), “Left lateral tongue” (LLT), “Palate,” and “Floor of the mouth” (FM) showed the highest concordance, reaching 100%. In contrast, areas such as the “Lips” also impacted overall concordance similarly to OM.

**Table 6:** Agreement between the gold standard and the four remote examiners by region in SOM.

Area	Agreement	Agreement	Agreement	Agreement	Mean Agreement
	PO X EX1	PO X EX2	PO X EX3	PO X EX4	(95%CI)
	(95%CI)	(95%CI)	(95%CI)	(95%CI)	(95%CI)
Lips	1.00	1.00	0.37	0.65	0.76
	(1–1)	(1–1)	(-0.013–0.76)	(0.019–1)	(0.48–1)
	p ≈ 0.000000121	p ≈ 0.000000121	p = 0.011	p = 0.000241	
RBM	96.4*	96.4*	82.1*	96.4*	0.93
	(89.6–100)	(89.6–100)	(67.9–96.3)	(89.6–100)	(0.87–0.99)
LBM	100*	100*	96.4	100*	0.99
	(100–100)	(100–100)	(89.6–100)	(100–100)	(0.98–1)
TD	1	1	0.34	0,34	0.67
	(1–1)	(1–1)	(-0.23–0.92)	(-0.23–0.92)	(0.337–1)
	p ≈ 0.000000121	p ≈ 0.000000121	p = 0.0623	p = 0.0623	
RLT	100*	100*	100*	100*	100*
	(100–100%)	(100–100%)	(100–100%)	(100–100%)	(100–100%)
LLT	100*	100*	100*	100*	100*
	(100–100)	(100–100	(100–100)	(100–100)	(100–100)
Palate	1.00	1.00	1.00	1.00	1
	(1–1)	(1–1)	(1–1)	(1–1)	(1–1)
	p ≈ 0.000000121	p ≈ 0.000000121	p ≈ 0.000000121	p ≈ 0.000000121	
FM	100*	100*	100*	100*	100*
	(100–100)	(100–100)	(100–100)	(100–100)	(100–100)
Mean	0.99	0.99	0.68	0.85	0.89
	(0.99–1)	(0.99–1)	(0.48–0.88)	(0.68–1)	(0.78–1)

RBM: right buccal mucosa; LBM: left buccal mucosa; TD: tongue dorsum; RLT: right lateral tongue; LLT: left lateral tongue; FM: floor of the mouth; G0: gold standard; Ex1: examiner 1; Ex2: examiner 2; Ex3: examiner 3; Ex4:examiner 4; * = Value calculated by simple agreement, derived from the proportion of correct responses among the evaluators relative to the gold standard due to the absence of true positive cases. Confidence intervals were calculated using the mean limits; C: confidence interval.

Examiner 3 also showed consistently lower concordance in this outcome, influencing the overall mean. Wide confidence intervals in some cases suggest variability in the examiners’ assessments, mainly due to the limited sample size per region analyzed (n = 28). These findings reinforce what was also observed in OM, indicating that although remote assessment is feasible, examiner alignment is essential to reduce inter-examiner variability.

## Discussion

Monitoring oral health conditions during treatment of pediatric and adolescent cancer can become unfeasible, as patients often travel back to their homes, generally located far from treatment centers.^25^ In this context, the Telepediatric Dentistry in Oncology (TON) application was developed to enable remote monitoring of pediatric cancer patients through photographic examinations, in addition to promoting oral health education for the entire family unit.^18^

Photographs obtained via mobile applications have proven useful in dental practice, being employed in oral cancer detection^8^ and in discussing clinical situations among professionals^12^, promoting greater efficiency in health services.^3^ The results of the present study indicate satisfactory performance of the photographs obtained through TON, all taken with 13-megapixel cameras, whose acceptable quality likely prevented image exclusion due to focus or lighting issues, and which is in agreement with studies indicating that 8-megapixel cameras are already adequate for image-based diagnosis.^3,26^

Considering that this study is preliminary and used images captured exclusively by trained researchers, clinical application will depend on the ability of caregivers, or even patients, to produce photos of sufficient quality in terms of focus and lighting. This scenario is planned to be tested in the future with TON users.

A 2025 study^27^ states that evaluations performed by caregivers using smartphones can be feasible, although challenges related to lighting, focus, and camera positioning may influence diagnostic accuracy. Thus, the accuracy of photos taken by parents with smartphones was useful for screening in teledentistry programs, expanding the possibility of remote monitoring.

No false negatives for OM or SOM were observed in the different sites examined, reinforcing that image quality was adequate. However, the reduced performance of examiner 3, along with wider confidence intervals, highlights the importance of continuous calibration and training to minimize inter-examiner variability and increase the robustness of remote assessments. The results of this preliminary study reinforce this point.

As expected, SOM lesions were easier to diagnose due to their more advanced stage and the presence of ulceration, reflected in higher accuracy compared to OM. The low agreement for OM may also be explained by the subtlety of early lesions, with a higher occurrence of false positives for this outcome, suggesting the need to standardize photographic protocols, especially since different smartphones with varying characteristics will be employed when the tool is used by caregivers. Additional training of evaluators can improve accuracy in future studies, with remote examiners ensuring uniformity of assessments.

The remote examination detected all OM/SOM cases diagnosed in-person, with 100% sensitivity across all sites, corroborating the ability of remote examinations using TON photographs to identify early changes. However, it is important to consider the clinical consequences of false-positive and false-negative results: false positives may lead to unnecessary interventions, while false negatives (which were not observed in the results of this study) could delay treatment, highlighting the need for clear protocols and careful image interpretation.

Considering the false positives in sites of higher prevalence, such as lips and buccal mucosa, the remote tool offers advantages, allowing evaluation at specific times and focus points with the possibility of image enlargement, facilitating examination. This approach is supported by evidence that teledentistry can complement clinical assessment, enabling early monitoring of oral conditions, including dental caries.^11,28-31^

Changes in therapeutic protocols, such as photobiomodulation protocols, can be enhanced by remote examination, which, according to the results, enables early detection of lesions not clinically observed. Low-level laser treatment is considered the most recommended intervention for oral mucositis, and therapeutic applications can be initiated sooner with early diagnosis.^32^ Thus, integrating TON into clinical practice could support earlier and more precise interventions.

Despite its contributions, the study has limitations. Although it is a pilot, the small sample size stands out. While this is justified by the vulnerability of the patients who may experience mortality, hospital biosafety restrictions, and short hospitalization periods, the results presented herein should be interpreted with caution. However, it is common knowledge that most studies with pediatric oncology patients are relatively small in size, partly because these diseases are rare.^33,34^ Additionally, our sample was obtained from a single cancer center. Thus, it is difficult to control all variables related to chemotherapy treatment and patient-related factors, and there is a practical challenge in obtaining a sufficient number of participants due to the low incidence of childhood cancer.

Unfortunately, loss to follow-up is frequently observed in research with cancer patients. Moreover, lack of statistical power is a common problem in cancer studies due to small sample sizes,^35^ as mentioned previously. Nevertheless, we highlight that the power of this study achieved over 80%, despite the small sample.

It is important to note that this is the first study to demonstrate the accuracy of an application for diagnosing oral mucositis in pediatric cancer patients, and the findings provide important preliminary evidence on the feasibility of telemonitoring in a complex clinical context such as oncology.

It is recommended to increase the sample size, standardize photographic protocols if photographs are taken by more than one person (e.g., app users), and use multiple devices for future research. Additionally, enhancing evaluator training with continuous assessment of observed perceptions is essential. Thus, the TON remote examination can serve as a teledentistry tool which safely contributes to clinical management in pediatric oncology patients when used by caregivers.

## Conclusion

The results suggest that the index test based on remote examinations using photographs is effective in identifying oral mucositis, even in its non-severe form. The absence of false negatives reinforces the potential applicability of this method.

Although factors which may affect generalization of the results should be considered, such as sample representativeness, examination losses, and the age range of participants, the effectiveness of the method when used by caregivers should be explored in future research. Thus, the evaluated tool shows potential for use in remote dental monitoring of pediatric cancer patients.

## Data Availability

The datasets generated during and/or analyzed during the current study are available from the corresponding author on reasonable request.
